# Optimum estimator in simple random sampling using two auxiliary attributes with application in agriculture, fisheries and education sectors

**DOI:** 10.1016/j.mex.2022.101915

**Published:** 2022-11-09

**Authors:** Monika Saini, Bhatt Ravi Jitendrakumar, Ashish Kumar

**Affiliations:** Department of Mathematics & Statistics, Manipal University Jaipur, Jaipur 303007, India

**Keywords:** Finite population, Auxiliary attribute, Simple random sampling, Bias, Mean squared error

## Abstract

In modern age of information technology, data is available everywhere in huge amount. Every sector generates lot of data every day. The investigation of each unit of data is not feasible due to limited resources like time, labor, and cost. In such situations, survey sampling is recommended to draw the information about the population parameters. Therefore, the main objective of present study is to develop an estimation method for obtaining the information about population parameter. We propose an optimum estimator for enhanced estimation of population mean in simple random sampling by utilizing the information of the two auxiliary attribute. The expression for bias, mean squared error (MSE) and minimum mean squared error of the proposed estimator are derived up to the first order of approximation and it is shown that the proposed estimator under derived conditions perform better than the existing estimators theoretically. Four population are demonstrated to assess the performance as well as applicability of the proposed estimator. The percentage relative efficiency (PRE) of proposed estimator for all the populations is 209.533, 163.852, 210.398 and 340.578, respectively. The numerical illustrations confirm that the proposed estimator dominates over the existing estimators.

• The main objective of present study is to propose a new estimator/method for estimation of population mean using two auxiliary attributes under simple random sampling.
• The bias and mean square error of the proposed estimator/method is derived and compared with the existing estimators to compare the efficiency theoretically.
• Applications of the proposed method/estimator is highlighted using thorough the real data sets of various sectors.

## Graphical abstract

**Figure:**
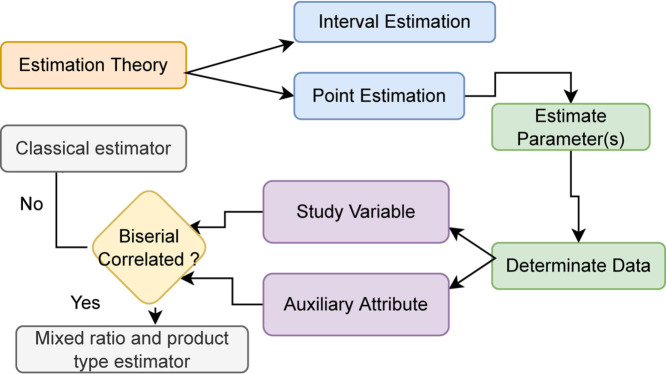

Specifications table

**Table:** 

Subject Area:	Mathematical Statistics
More specific subject area:	Sample Surveys
Method name:	Optimum estimator for estimating population mean using two auxiliary attributes
Name and references of original method:	The proposed method is motivated by following references: • Ahmad, S., Arslan, M., Khan, A., & Shabbir, J. (2021). A generalized exponential-type estimator for population mean using auxiliary attributes. *Plos one, 16*(5), e0246947. • Zaman, T., & Kadilar, C. (2019). Novel family of exponential estimators using information of auxiliary attribute. *Journal of Statistics and Management Systems, 22*(8), 1499–1509 • Lu, J. (2017). Efficient estimator of a finite population mean using two auxiliary variables and numerical application in agricultural, biomedical, and power engineering. *Mathematical Problems in Engineering*.
Resource availability:	Data utilized in the analysis is available in public domain.

## Background

In sample surveys, it is well documented in Cochran [20] that the use of supplementary information provided by auxiliary variables or attributes is frequently used for increasing the precision of the estimators by taking the advantages of correlation between the study variable and auxiliary variable. Regression, ratio, and product estimators are good examples in this perspective. Though Cochran [1] investigated that ratio estimator is best suited when the study and auxiliary variable are highly positively correlated whereas in case of highly negative correlated variables, product method of estimation is better. In many real-life situations, the study variable is not always quantitative in nature. The responses recorded from respondents are qualitative in such situations the recorded information is called attributes. Several studies like Shabbir and Gupta [22] and Abd-Elfattah et al. [23] have been conducted to improve the precision of the estimator by utilizing the auxiliary attributes having applications in agriculture, health science, fisheries, power engineering etc. It is also investigated that use of more than one auxiliary attribute enhance the efficiency of the estimator. Singh et al. [2] used auxiliary attribute information for establishment of ration estimators in simple random sampling (SRS). The bias and mean square error of the estimator has been computed for existing data set available in literature. It is developed as a modified estimator of Koyuncu and Kadilar [21] estimator and proved that it outperformed the existing estimators. Singh and Kumar [4] used auxiliary information to estimate improved regression estimator for SRS. Here the auxiliary information is qualitative, and concept of non-response incorporated in estimation. Malik and Singh [3] initiated the use of two auxiliary variables in estimation of population mean and proposed enhanced estimators. Here, auxiliary information available in qualitative form and this estimator performed better than simple regression estimator. Ekpenyong and Enang [5] suggested better exponential estimators in SRS for estimating population mean. The concept of simple random sampling without replacement used for development of estimator. Lu [6] explored the applications of estimators developed under auxiliary information in agriculture and power engineering sectors. The estimator compared with regression estimator and other existing estimators and proved efficient. Zaman and Kadilar [7] utilized auxiliary information for development of a novel family of exponential estimators. Ahmad et al. [8] carried out the generalization of exponential ratio estimators under auxiliary estimators. The estimator was exponential-based estimator while estimator proposed by us is mixed type estimator while estimator proposed in present study is a mixture of simple, ratio and product estimators. Mahajan et al. [9] explored the applications of estimation and sample surveys in agriculture and health sciences. Kumar and Saini [10] suggested a predictive approach to estimate the population mean under auxiliary attribute. Yunusa et al. [11] utilized auxiliary variables in development of regression type estimators to estimation the population mean. Rather et al. [24] used auxiliary information for development of a mixed exponential ratio type estimator for estimating the population mean. The simple random sampling and double sampling techniques utilized for selection of the sample. Zaman et al. [25] proposed an exponential type estimator for assessing and estimating the COVID-19 risk in various countries. Two multivariate families of exponential type estimators proposed by utilizing the information on two auxiliary variables. Many authors like Wayangkau et al. [18], Waheeb et al. [15], Rajak [16], and Jabal et al. [17] discussed some other data analysis techniques for analysis of agricultural information. The above cited literature motivates to explore the applicability of two auxiliary attributes in estimation of mean of various populations associated with agriculture, fisheries, and education sectors. The primary goal of this paper is to propose a novel optimum estimator for estimating finite population mean using auxiliary attributes. The expressions for the bias and mean square error (MSE) of the proposed estimator are inferred up to the first order of approximation. On the bases of theoretical and numerical comparisons, we demonstrate that the proposed estimator is more efficient than existing estimators.

## Material and methods

Consider $\gamma =(\gamma_{1},\;\gamma_{2},\;\gamma_{3},\;\ldots .,\;\gamma_{N})$ be a finite population of size N. we draw a sample of size n (with *n*<*N*) units from γ using simple random sample without replacement (SRSWOR). Let *y_i_* be the study variable and *ẟ_i_* be the characteristics of the auxiliary attributes i.e. *ẟ_i_* *= 1* if the *i*th unit possess attribute and *ẟ_i_ = 0,* otherwise. Let $G=\sum_{i=1}^{N}\delta_{i}\;$be the total number of units in the population possessing attributes *ẟ_i_* and $g=\sum_{i=1}^{n}\delta_{i}\;$be the total number of units in the sample possessing attributes *ẟ_i_*. Let D=(G/N) be the proportion of units in the population and d=(g/n) be the population of units in the sample. Let $\bar{Y}=\frac{\sum_{i=1}^{N}y_{i}}{N}\;$and $\bar{y}=\frac{\sum_{i=1}^{n}y_{i}}{n}\;$be the population and sample mean of the study variable. Let D_1_ and D_2_ be the population proportion of the auxiliary attributes and sample proportion of auxiliary attributes is denoted by d_1_ and d_2_. Let $S_{y}^{2}=\frac{\sum_{i=1}^{N}{\left(y_{i}-\bar{Y}\right)}^{2}}{N-1}\;$be the population variance of the study variable y. Let $S_{d_{1}}^{2}=\frac{\sum_{i=1}^{N}{\left(d_{1}-D_{1}\right)}^{2}}{N-1}\;$and $S_{d_{2}}^{2}=\frac{\sum_{i=1}^{N}{\left(d_{2}-D_{2}\right)}^{2}}{N-1}\;$respectively be the population variance of the auxiliary attributes d_1_ and d_2_. Let $C_{y}=\frac{S_{y}}{\bar{Y}}$ be the coefficient of variation of the study variable y. Let $C_{d1}=\frac{S_{d1}}{D_{1}}$ and $C_{d2}=\frac{S_{d2}}{D_{2}}$ be the coefficient of variation of the auxiliary d_1_ and d_2_. Let $S_{yd_{j}}=\frac{\sum_{i=1}^{N}\left(y_{i}-\bar{Y}\right)\left(d_{j}-D_{j}\right)}{N-1}\;$be the population covariance between the study variable y and the auxiliary attributes $d_{j}\left(j=1,2\right).$ Let $S_{d_{1}d_{2}}=\frac{\sum_{i=1}^{N}\left(d_{1}-D_{1}\right)\left(d_{2}-D_{2}\right)}{N-1}\;$be the population covariance between the auxiliary attributes d_1_ and d_2_. Let $\rho_{yd_{j}}=\frac{S_{yd_{j}}}{S_{y}S_{d_{j}}}$ be the population point bi-serial correlation coefficient between the study variable y and the auxiliary attribute $d_{j}\left(j=1,2\right).\;$Let $\pi_{0}=\frac{\bar{y}-\bar{Y}}{\bar{Y}}$; $\pi_{1}=\frac{d_{1}-D_{1}}{D_{1}}$ and $\pi_{2}=\frac{d_{2}-D_{2}}{D_{2}}$ be the error terms such that $E\left[e_{i}\right]=0\;\left(i=0,1,2\right)$, $E\left[\pi_{0}^{2}\right]=\xi C_{y}^{2},\;E\left[\pi_{1}^{2}\right]=\xi C_{d_{1}}^{2},\;E\left[\pi_{2}^{2}\right]=\xi C_{d_{2}}^{2}$,$E\left[\pi_{0}\pi_{1}\right]=\xi \rho_{yd_{1}}C_{y}C_{d_{1}},\;E\left[\pi_{0}\pi_{2}\right]=\xi \rho_{yd_{2}}C_{y}C_{d_{2}}$and$\;E\left[\pi_{1}\pi_{2}\right]=\xi \rho_{d_{1}d_{2}}C_{d_{1}}C_{d_{2}}\;$where $\xi =(\frac{1}{n}-\frac{1}{N})$ and $f=\frac{n}{N}$ .

The stepwise framework of proposed method is described as follows:

Step 1: Consider a finite population of size N.
Step 2: Select a random sample of size n from the population using simple random sampling without replacement.
Step 3: Observe *y_i_* and *ẟ_i_* from sampling units.
Step 4: Define the expressions for population and sample characteristics.
Step 5: Propose the estimator for estimating population mean using two auxiliary attributes and derive its properties.
Step 6: Compare the proposed estimator with the existing estimators theoretically and numerically.

### Existing estimators

#### Unbiased estimator (${\hat{\mu }}_{0}$)

The most widely used estimator discussed by Cochran [1] of population mean $\bar{Y}\;$of the study variable, is given by

(1) $${\hat{\mu }}_{0=}\bar{y},$$

sample mean is an unbiased estimator of population mean and upto the first order of approximation the variance or MSE is given by

(2) $$V\left({\hat{\mu }}_{0}\right)=V\left(\bar{y}\right)=\xi {\bar{Y}}^{2}c_{y}^{2}\;.$$

#### Naik and Gupta (${\hat{\mu }}_{1}$)

Naik and Gupta [12] proposed the following ratio estimator of population mean $\bar{Y}\;$when the population proportion $D_{1}$ of auxiliary attribute is known

(3) $${\hat{\mu }}_{1}=\bar{y}\left(\frac{D_{1}}{d_{1}}\right),$$

The bias and MSE of ${\hat{\mu }}_{1}$ to the first order of approximation is given by,

$$Bias\left({\hat{\mu }}_{1}\right)≅\xi \bar{Y}\left[c_{d1}^{2}-\rho_{yd1}c_{y}c_{d1}\right],$$

and

(4) $$MSE\left({\hat{\mu }}_{1}\right)≅\xi {\bar{Y}}^{2}\left[c_{y}^{2}+c_{d1}^{2}-2\rho_{yd1}c_{y}c_{d1}\right]\;.$$

#### Naik and Gupta (${\hat{\mu }}_{2}$)

Naik and Gupta [12] proposed the following product estimator of population mean $\bar{Y}\;$when the population proportion $D_{1}$ of auxiliary attribute is known

(5) $${\hat{\mu }}_{2}=\bar{y}\left(\frac{d_{1}}{D_{1}}\right),$$

The bias and MSE of ${\hat{\mu }}_{2}$, to the first order of approximation is given by

$$Bias\left({\hat{\mu }}_{2}\right)≅\bar{Y}\xi \left[\rho_{yd1}c_{y}c_{d1}\right],$$

and

(6) $$MSE\left({\hat{\mu }}_{2}\right)≅\xi {\bar{Y}}^{2}\left[c_{y}^{2}+c_{d1}^{2}+2\rho_{\text{yd}1}c_{y}c_{d1}\right].$$

#### Singh et al. (${\hat{\mu }}_{3}$)

Singh et al. [2] suggested an exponential type ratio estimator for estimating population mean $\bar{Y}\;$using the population proportion $D_{1}$ of auxiliary attribute is known

(7) $${\hat{\mu }}_{3}=\bar{y}\;exp\left[\frac{D_{1}-d_{1}}{D_{1}+d_{1}}\right],$$

The bias and MSE of ${\hat{\mu }}_{3}$, to the first order of approximation,

$$Bias\left({\hat{\mu }}_{3}\right)≅\bar{Y}\xi \left[\frac{3}{8}c_{d1}^{2}-\frac{1}{2}\rho_{yd1}c_{y}c_{d1}\right],$$

and

(8) $$MSE\left({\hat{\mu }}_{3}\right)≅\xi {\bar{Y}}^{2}\left[c_{y}^{2}+\frac{1}{4}c_{d1}^{2}-\rho_{yd1}c_{y}c_{d1}\right]\;.$$

#### Singh et al. (${\hat{\mu }}_{4}$)

Singh et al. [2] suggested the following product estimators for estimating population mean $\bar{Y}\;$when the population proportion $D_{1}$ of auxiliary attribute is known

(9) $${\hat{\mu }}_{4}=\bar{y}\;exp\left[\frac{d_{1}-D_{1}}{d_{1}+D_{1}}\right],$$

Similarly, the bias and MSE of ${\hat{\mu }}_{4}$, is given by

$$Bias\left({\hat{\mu }}_{4}\right)≅\frac{1}{2}\bar{Y}\xi \left[\rho_{yd1}c_{y}c_{d1}-\frac{1}{4}c_{d1}^{2}\right],$$

and

(10) $$MSE\left({\hat{\mu }}_{4}\right)≅\frac{1}{2}{\bar{Y}}^{2}\xi \left[c_{y}^{2}+\frac{1}{4}c_{d1}^{2}+\rho_{yd1}c_{y}c_{d1}\right].$$

#### Kumar and Bhougal (${\hat{\mu }}_{5}$)

Kumar and Bhougal [13] proposed an exponential type of ratio-product estimator for estimating population mean $\bar{Y}\;$when the population proportion $D_{1}$ of auxiliary attribute is known

(11) $${\hat{\mu }}_{5}=\bar{y}\;\left[\alpha \;exp\left(\frac{D_{1}-d_{1}}{D_{1}+d_{1}}\right)+\left(1-\alpha \right)\;exp\left(\frac{D_{1}-d_{1}}{D_{1}+d_{1}}\right)\right],$$

where α is unknown constant.

The bias and MSE of ${\hat{\mu }}_{5}$ to the first order of approximation is given by

$$Bias\left({\hat{\mu }}_{5}\right)≅\bar{Y}\xi \left[\frac{1}{8}\left(4\alpha -1\right)c_{d1}^{2}-\left(\alpha -\frac{1}{2}\right)\rho_{yd1}c_{y}c_{d1}\right],$$

and

(12) $$MSE\left({\hat{\mu }}_{5}\right)≅\xi {\bar{Y}}^{2}c_{y}^{2}\left[1-\rho_{yd1}^{2}\right]\;.$$

The optimum value of α is given by

$$\alpha_{opt}≅\frac{1}{2}+\rho_{yd1}\frac{c_{y}}{c_{p1}}.$$

#### Singh and Kumar (${\hat{\mu }}_{6}$)

Singh and Kumar [4] suggested ratio estimator for estimating population mean $\bar{Y}\;$when the population proportion $D_{1}$ and $D_{2\;}$of auxiliary attribute are known

(13) $${\hat{\mu }}_{6}=\bar{y}\;\left(\frac{D_{1}}{d_{1}}\right)\left(\frac{D_{2}}{d_{2}}\right),$$

The bias and MSE of ${\hat{\mu }}_{6}$ to the first order of approximation are given by

$$Bias\left({\hat{\mu }}_{6}\right)≅\bar{Y}\xi \left[c_{d1}^{2}+c_{d2}^{2}+\rho_{d1d2}c_{d1}c_{d2}-\rho_{yd1}c_{y}c_{d1}-\rho_{yd2}c_{y}c_{d2}\right],$$

and

(14) $$MSE\left({\hat{\mu }}_{6}\right)≅\xi {\bar{Y}}^{2}\left[c_{y}^{2}+c_{d1}^{2}+c_{d2}^{2}-2\;\left(\rho_{yd1}c_{y}c_{d1}-\rho_{yd1}c_{y}c_{d1}-\rho_{yd2}c_{y}c_{d2}\right)\right].$$

#### Singh and Kumar (${\hat{\mu }}_{7}$)

Singh and Kumar [4] suggested product estimator for estimating population mean $\bar{Y}\;$when the population proportion $D_{1}$ and $D_{2\;}$of auxiliary attribute are known

(15) $${\hat{\mu }}_{7}=\bar{y}\;\left(\frac{d_{1}}{D_{1}}\right)\left(\frac{d_{2}}{D_{2}}\right),$$

The bias and MSE of ${\hat{\mu }}_{7}$ to the first order of approximation are given by

$$Bias\left({\hat{\mu }}_{7}\right)≅\bar{Y}\xi \left[\rho_{d1d2}c_{d1}c_{d2}-\rho_{yd1}c_{y}c_{d1}+\rho_{yd2}c_{y}c_{d2}\right],$$

(16) $$MSE\left({\hat{\mu }}_{7}\right)≅\xi {\bar{Y}}^{2}\left[c_{y}^{2}+c_{d1}^{2}+c_{d2}^{2}+2\;\left(\rho_{yd1}c_{y}c_{d1}-\rho_{yd1}c_{y}c_{d1}+\rho_{yd2}c_{y}c_{d2}\right)\right].$$

#### Ahmed et al. (${\hat{\mu }}_{8}$)

Ahmed et al. [8] proposed a generalized class of factor type of estimators for estimating population mean $\bar{Y}\;$when the population proportion $D_{1}$ and $D_{2\;}$of auxiliary attribute are known is given by

(17) $${\hat{\mu }}_{8}=\bar{y}\left[\exp\left(\frac{S_{1}-M_{1}}{S_{1}+M_{1}}\right)\exp\left(\frac{S_{2}-M_{2}}{S_{2}+M_{2}}\right)\right]$$

where,

$S_{1}=\left(A_{1}+C_{1}\right)D_{1}+FB_{1}d_{1}$, $S_{2}=\left(A_{2}+C_{2}\right)D_{2}+FB_{2}d_{2}$, $M_{1}=\left(A_{1}+FB_{1}\right)D_{1}+C_{1}d_{1}$, $M_{2}=\left(A_{2}+FB_{2}\right)D_{2}+C_{2}d_{2}$, $A_{i}=\left(K_{i}-1\right)\left(K_{i}-2\right)$, $B_{i}=\left(K_{i}-1\right)\left(K_{i}-4\right)$, and $C_{i}=\left(K_{i}-2\right)\left(K_{i}-3\right)\left(K_{i}-4\right)$*.*

The bias and MSE of ${\hat{\mu }}_{8}$ to the first order of approximation are given by

$$Bias\left({\hat{\mu }}_{8}\right)=\bar{y}\xi \left[\frac{1}{2}\sigma_{1}\rho_{yd1}C_{y}C_{d1}+\frac{1}{2}\sigma_{2}\rho_{yd2}C_{y}C_{d2}+\frac{1}{8}C_{d1}^{2}\left(\sigma_{1}^{2}-2\sigma_{1}v_{1}\right)+\frac{1}{8}C_{d2}^{2}\left(\sigma_{2}^{2}-2\sigma_{2}v_{2}\right)\right],$$

where $v_{1}=\frac{FB_{1}+C_{1}}{A_{1}+FB_{1}+C_{1}}$ and $v_{2}=\frac{FB_{2}+C_{2}}{A_{2}+FB_{2}+C_{2}}$ .

The minimum MSE of $({\hat{\mu }}_{8})\;\text{to}\;\text{the}\;\text{first}\;\text{order}\;\text{of}\;\text{approximation}$ is given by

(18) $$\mathrm{MSE}\left({\hat{\mu }}_{8}\right)≅{\bar{y}}^{2}C_{y}^{2}\left[1-R_{\mathrm{yd}1d2}^{2}\right],$$

where

$R_{yd1d2}^{2}=\frac{\rho_{yd1}^{2}+\rho_{yd2}^{2}-2\rho_{yd1}\rho_{yd2}\rho_{d1d2}}{1-\rho_{d1d2}^{2}}$,

The optimum value of $\sigma_{10pt}$ and $\sigma_{20pt}$ are

$$\sigma_{10pt}=\frac{2C_{y}\left(\rho_{yd1}-\rho_{d1d2}\rho_{yd2}\right)}{C_{d1}\left(\rho_{d1d2}^{2}-1\right)}\;\text{and}\;\sigma_{20pt}=\frac{2C_{y}\left(\rho_{yd2}-\rho_{d1d2}\rho_{yd1}\right)}{C_{d2}\left(\rho_{d1d2}^{2}-1\right)}.$$

## Proposed estimator & its properties

Motivated by Singh and Espejo [14] and Lu [6], adopting the same procedure we proposed as estimator for estimating population mean by utilizing two auxiliary attributes as

(19) $$\left({\hat{\mu }}_{pop}\right)=\frac{\omega_{1}\bar{y}+\omega_{2}\left(D_{1}-d_{1}\right)+\omega_{3}\left(D_{2}-d_{2}\right)}{4}\left(\frac{D_{1}}{d_{1}}+\frac{d_{1}}{D_{1}}\right)\left(\frac{D_{2}}{d_{2}}+\frac{d_{2}}{D_{2}}\right)$$

To obtain the bias and MSE of proposed estimator, applying the error approximation into Eq. (17), in terms of π’s, the proposed estimator can be expressed as

(20) $${\hat{\mu }}_{prop}=\frac{\omega_{1}\bar{Y}\left(\pi_{0}+1\right)-\omega_{2}D_{1}\pi_{1}-\omega_{3}D_{2}\pi_{2}}{4}\left(\frac{1}{\pi_{1}+1}+\pi_{1}+1\right)\left(\frac{1}{\pi_{2}+1}+\pi_{2}+1\right)$$

Expanding the right-hand side of [20], we get

(21) $${\hat{\mu }}_{prop}=\frac{\omega_{1}\bar{Y}\left(\pi_{0}+1\right)-\omega_{2}D_{1}\pi_{1}-\omega_{3}D_{2}\pi_{2}}{4}\left[\pi_{1}+1+\left(1-\pi_{1}+\pi_{1}^{2}+\cdots \right)\right]\left[\pi_{2}+1+\left(1-\pi_{2}+\pi_{2}^{2}+\cdots \right)\right].$$

In Eq. (21) we will neglect the terms of π’s, power having greater than two, we get

(22) $${\hat{\mu }}_{prop}-\bar{Y}≅\left(\omega_{1}-1\right)\bar{Y}+\omega_{1}\bar{Y}\pi_{0}-\omega_{2}D_{1}\pi_{1}-\omega_{3}D_{2}\pi_{2}+\frac{1}{2}\omega_{1}\bar{Y}\pi_{1}^{2}+\frac{1}{2}\omega_{1}\bar{Y}\pi_{2}^{2},$$

**Fig. 1:**
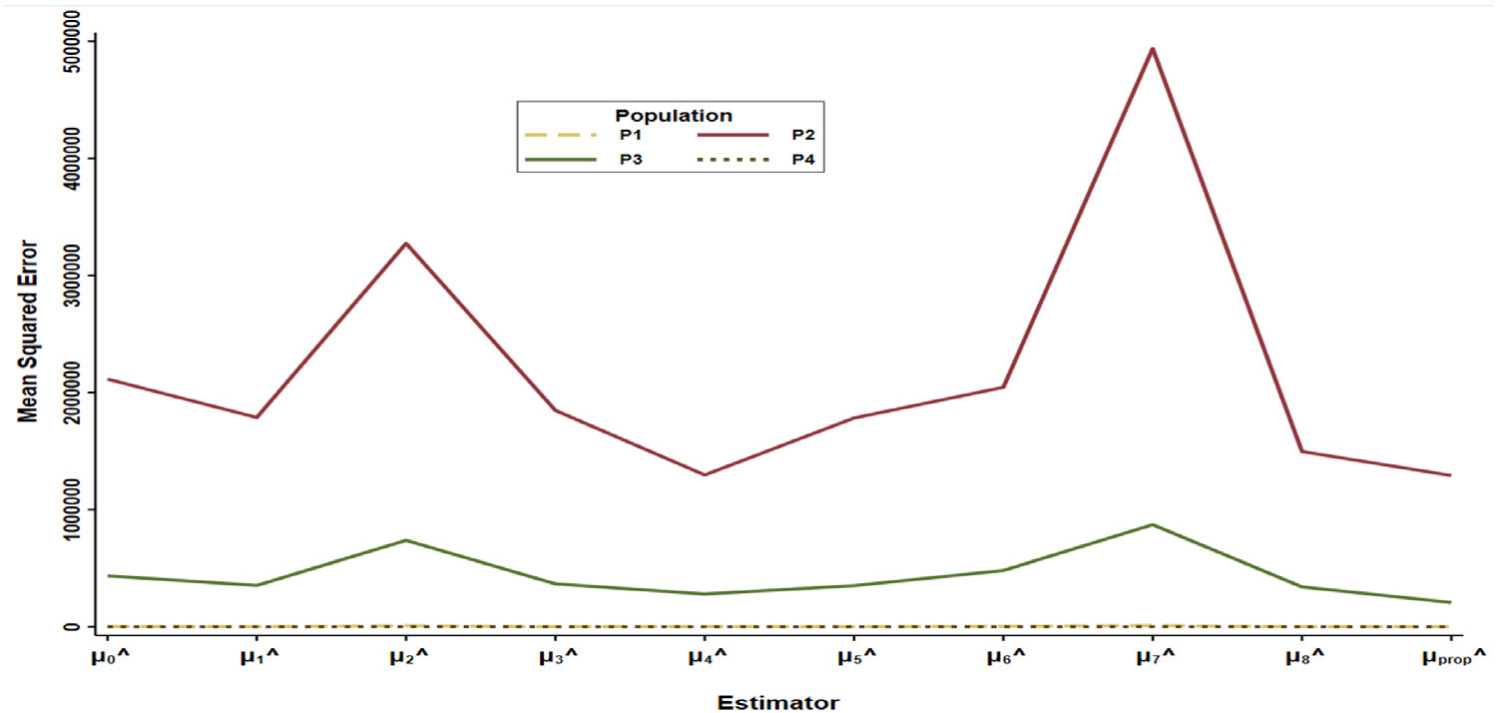
Graphical representation of estimators w.r.t to their MSE.

To get the bias of the proposed estimator, we need to take expectation on both the sides of (22), hence we will get the bias of the proposed estimator up to the first order of approximation (Fig. 1).

$$E\left({\hat{\mu }}_{prop}-\bar{Y\;}\right)≅\left(\omega_{1}-1\right)\bar{Y}+\omega_{1}\bar{Y}E\left(\pi_{0}\right)-\omega_{2}D_{1}E\left(\pi_{1}\right)-\omega_{3}D_{2}E\left(\pi_{2}\right)+\frac{1}{2}\omega_{1}\bar{Y\;}E\left(\pi_{1}^{2}\right)+\frac{1}{2}\omega_{1}\bar{Y}E\left(\pi_{2}^{2}\right).$$

The bias of the proposed estimator is given by

$$Bias\left({\hat{\mu }}_{prop}\right)=\left[E\left({\hat{y}}_{pr}\right)-\bar{Y}\right]≅\bar{Y}\left[\left(\omega_{1}-1\right)+\frac{1}{2}\xi \omega_{1}C_{d1}^{2}+\frac{1}{2}\xi \omega_{1}C_{d2}^{2}\right].$$

Now for deriving the MSE of the proposed estimator, let us square both sides of (22), neglecting terms π’s having power greater than two, and taking expectation on both sides, after simplification we get,

(23) $$\begin{array}{cc} & MSE\left({\hat{\mu }}_{prop}\right)=E{\left({\hat{\mu }}_{prop}-\bar{Y}\right)}^{2},\\ & ≅{\bar{Y}}^{2}[{\left(\omega_{1}-1\right)}^{2}-\xi \omega_{1}\left(C_{d1}^{2}+C_{d2}^{2}\right)+\xi \omega_{1}\left(C_{d1}^{2}+C_{d2}^{2}+C_{y}^{2}\right)-2\xi \omega_{1}\bar{Y}C_{y}\left(\omega_{2}C_{d1}\rho_{yd1}D_{1}+\omega_{3}C_{d2}\rho_{yd2}D_{2}\right)\\ & \;+\;\xi \left(\omega_{2}^{2}C_{d1}^{2}D_{1}^{2}+2C_{d1}C_{d2}\omega_{2}\omega_{3}\rho_{d1d2}D_{d1}D_{d2}+\omega_{3}^{2}C_{d2}^{2}D_{2}^{2}\right)]. \end{array}$$

To obtain the optimum value of $\omega_{1},\omega_{2}\;and\;\omega_{3}$, we partially differentiate the Eq. (23) with respect to $\omega_{1},\omega_{2}\;and\;\omega_{3}$ and put it equal to zero, the optimum values of $\omega_{1},\omega_{2}\;and\;\omega_{3}$ are given by

$$\omega_{1}^{*}=\frac{\beta \left[2+\xi \left(C_{d1}^{2}+C_{d2}^{2}\right)\right]}{2\left[\beta +C_{y}^{2}\xi \alpha +\beta \xi \left(C_{d1}^{2}+C_{d2}^{2}\right)\right]},$$

$$\omega_{2}^{*}=\frac{C_{y}\bar{Y}\left(\rho_{yd1}-\rho_{d1d2}\;\rho_{yd2}\right)\left[2+\xi \left(C_{d1}^{2}+C_{d2}^{2}\right)\right]}{2C_{d1}D_{1}\left[\beta +C_{y}^{2}\xi \alpha +\beta \xi \left(C_{d1}^{2}+C_{d2}^{2}\right)\right]},$$

$$\omega_{3}^{*}=\frac{C_{y}\bar{Y}\left(\rho_{yd2}-\rho_{d1d2}\;\rho_{yd1}\right)\left[2+\xi \left(C_{d1}^{2}+C_{d2}^{2}\right)\right]}{2C_{d2}D_{2}\left[\beta +C_{y}^{2}\xi \alpha +\beta \xi \left(C_{d1}^{2}+C_{d2}^{2}\right)\right]}.$$

The minimum MSE of ${\hat{\mu }}_{prop}$ can be shown as,

(24) $$MSE{\left({\hat{\mu }}_{prop}\right)}_{min}=\frac{\xi {\bar{Y}}^{2}\left[4C_{y}^{2}\alpha -\beta \xi {\left(C_{d1}^{2}+C_{d2}^{2}\right)}^{2}\right]}{4\left[\beta +C_{y}^{2}\xi \alpha +\beta \xi \left(C_{d1}^{2}+C_{d2}^{2}\right)\right]},$$

where $\alpha =1-\rho_{d1d2}^{2}-\rho_{yd1}^{2}+2\rho_{d1d2}\;\rho_{yd1}\;\rho_{yd2}-\rho_{yd2}^{2}$ and $\beta =1-\rho_{d1d2}^{2}$.

## Theoretical comparison of estimators

In this section we compare our proposed estimator with the exiting estimators discussed in Section 2, we will have the conditions as follows:

From Eqs. (2) and (24)

$$MSE\left({\hat{\mu }}_{0}\right)>MSE{\left({\hat{\mu }}_{prop}\right)}_{min}\;\text{if}\;\text{and}\;\text{only}\;\text{if}$$

$$c_{y}^{2}>\frac{\left[4C_{y}^{2}\alpha -\beta \xi {\left(C_{d1}^{2}+C_{d2}^{2}\right)}^{2}\right]}{4\left[\beta +C_{y}^{2}\xi \alpha +\beta \xi \left(C_{d1}^{2}+C_{d2}^{2}\right)\right]}\;.$$

From Eqs. (4) and (24)

$$MSE\left({\hat{\mu }}_{1}\right)>MSE{\left({\hat{\mu }}_{prop}\right)}_{min}\;\text{if}\;\text{and}\;\text{only}\;\text{if}$$

$$\left[c_{y}^{2}+c_{d1}^{2}-2\rho_{yd1}c_{y}c_{d1}\right]>\frac{\left[4C_{y}^{2}\alpha -\beta \xi {\left(C_{d1}^{2}+C_{d2}^{2}\right)}^{2}\right]}{4\left[\beta +C_{y}^{2}\xi \alpha +\beta \xi \left(C_{d1}^{2}+C_{d2}^{2}\right)\right]}$$

From Eqs. (6) and (24)

$$MSE\left({\hat{\mu }}_{2}\right)>MSE{\left({\hat{\mu }}_{prop}\right)}_{min}\;\text{if}\;\text{and}\;\text{only}\;\text{if}$$

$$\left[c_{y}^{2}+c_{d1}^{2}+2\rho_{yd1}c_{y}c_{d1}\right]>\frac{\left[4C_{y}^{2}\alpha -\beta \xi {\left(C_{d1}^{2}+C_{d2}^{2}\right)}^{2}\right]}{4\left[\beta +C_{y}^{2}\xi \alpha +\beta \xi \left(C_{d1}^{2}+C_{d2}^{2}\right)\right]}$$

From Eqs. (8) and (24)

$$MSE\left({\hat{\mu }}_{3}\right)>MSE{\left({\hat{\mu }}_{prop}\right)}_{min}\;\text{if}\;\text{and}\;\text{only}\;\text{if}$$

$$\left[c_{y}^{2}+\frac{1}{4}c_{d1}^{2}-\rho_{yd1}c_{y}c_{d1}\right]>\frac{\left[4C_{y}^{2}\alpha -\beta \xi {\left(C_{d1}^{2}+C_{d2}^{2}\right)}^{2}\right]}{4\left[\beta +C_{y}^{2}\xi \alpha +\beta \xi \left(C_{d1}^{2}+C_{d2}^{2}\right)\right]}$$

From Eqs. (10) and (24)

$$MSE\left({\hat{\mu }}_{4}\right)>MSE{\left({\hat{\mu }}_{prop}\right)}_{min}\;\text{if}\;\text{and}\;\text{only}\;\text{if}$$

$$\left[c_{y}^{2}+\frac{1}{4}c_{d1}^{2}+\rho_{yd1}c_{y}c_{d1}\right]>\frac{\left[4C_{y}^{2}\alpha -\beta \xi {\left(C_{d1}^{2}+C_{d2}^{2}\right)}^{2}\right]}{4\left[\beta +C_{y}^{2}\xi \alpha +\beta \xi \left(C_{d1}^{2}+C_{d2}^{2}\right)\right]}$$

From Eqs. (12) and (24)

$$MSE\left({\hat{\mu }}_{5}\right)>MSE{\left({\hat{\mu }}_{prop}\right)}_{min}\;\text{if}\;\text{and}\;\text{only}\;\text{if}$$

$$c_{y}^{2}\left[1-\rho_{yd1}^{2}\right]>\frac{\left[4C_{y}^{2}\alpha -\beta \xi {\left(C_{d1}^{2}+C_{d2}^{2}\right)}^{2}\right]}{4\left[\beta +C_{y}^{2}\xi \alpha +\beta \xi \left(C_{d1}^{2}+C_{d2}^{2}\right)\right]}$$

From Eqs. (14) and (24)

$$MSE\left({\hat{\mu }}_{6}\right)>MSE{\left({\hat{\mu }}_{prop}\right)}_{min}\;\text{if}\;\text{and}\;\text{only}\;\text{if}$$

$$\left[c_{y}^{2}+c_{d1}^{2}+c_{d2}^{2}-2\;\left(\rho_{yd1}c_{y}c_{d1}-\rho_{yd1}c_{y}c_{d1}+\rho_{yd2}c_{y}c_{d2}\right)\right]>\frac{\left[4C_{y}^{2}\alpha -\beta \xi {\left(C_{d1}^{2}+C_{d2}^{2}\right)}^{2}\right]}{4\left[\beta +C_{y}^{2}\xi \alpha +\beta \xi \left(C_{d1}^{2}+C_{d2}^{2}\right)\right]}$$

From Eqs. (16) and (24)

$$MSE\left({\hat{\mu }}_{7}\right)>MSE{\left({\hat{\mu }}_{prop}\right)}_{min}\;\text{if}\;\text{and}\;\text{only}\;\text{if}$$

$$\left[c_{y}^{2}+c_{d1}^{2}+c_{d2}^{2}+2\;\left(\rho_{yd1}c_{y}c_{d1}-\rho_{yd1}c_{y}c_{d1}+\rho_{yd2}c_{y}c_{d2}\right)\right]>\frac{\left[4C_{y}^{2}\alpha -\beta \xi {\left(C_{d1}^{2}+C_{d2}^{2}\right)}^{2}\right]}{4\left[\beta +C_{y}^{2}\xi \alpha +\beta \xi \left(C_{d1}^{2}+C_{d2}^{2}\right)\right]}$$

From Eqs. (18) and (24)

$$MSE\left({\hat{\mu }}_{8}\right)>MSE{\left({\hat{\mu }}_{prop}\right)}_{min}\;\text{if}\;\text{and}\;\text{only}\;\text{if}$$

$${\bar{y}}^{2}C_{y}^{2}\left[1-R_{yd1d2}^{2}\right]>\frac{\left[4C_{y}^{2}\alpha -\beta \xi {\left(C_{d1}^{2}+C_{d2}^{2}\right)}^{2}\right]}{4\left[\beta +C_{y}^{2}\xi \alpha +\beta \xi \left(C_{d1}^{2}+C_{d2}^{2}\right)\right]}$$

## Results and discussion

To examine the dominance and applicability of proposed estimator in simple random sampling, we considered four real data sets from agricultural, fishers and education sectors available in Ahmad et al. [8]. A similar kind of population for reference is given as Appendix A.

**Population 1**. (Source from education sector)

Let y represent the number of instructors, $d_{1}$ the total number of primary and secondary school students in Turkey in 2007, which was larger than 11,440.5, and $d_{2}$ the total number of primary and secondary school students in Turkey in 2008, which was greater than 333.1647. The population information about data set is given as:

$$\begin{array}{cc} & N=923,\;n=180,\;\bar{\;Y}=436.4346,\;D_{1}=2.6625,\;\;D_{2}=3.125,\;\rho_{yd1}=0.6904898,\\ & \rho_{yd2}=0.652149,\rho_{d1d2}=0.8465885,\;C_{d1}=1.826732,\;C_{d2}=1.641621,\;\;C_{y}=1.718333 \end{array}$$

**Population 2.** (Source from fishers’ sector)

Let y represent the expected catch by recreational marine fishermen in 1995, $d_{1}$ represent the percentage of fish captured larger than 1000 in 1993, and $d_{2}$ represent the percentage of fish caught greater than 2000 in 1994. The population information about data set is given as:

$$\begin{array}{cc} & N=69,\;n=14,\;\bar{Y}=4514.89,\;D_{1}=0.7391304,\;D_{2}=0.5507246,\;\rho_{yd1}=0.3966081,\\ & \rho_{yd2}=0.538047,\;\rho_{d1d2}=0.6577519,\;C_{d1}=0.5984409,\;C_{d2}=0.9098277,\;\;C_{y}=1.350 \end{array}$$

**Population 3.** (Source from agricultural sector)

For the 47 districts of Pakistan, let y represent the tobacco area production in hectares for the year 2009, $d_{1}$ represent the percentage of farms with tobacco cultivation areas greater than 500 ha for the year 2007, and $d_{2}$ represent the percentage of farms with tobacco cultivation areas greater than 800 ha for the year 2008. The population information about data set is given as:

$$\begin{array}{cc} & N=47,\;n=10,\;\bar{\;Y}=1004.447,\;D_{1}=0.4255319,\;D_{2}=0.3829787,\;\rho_{\text{yd}1}=0.4395989,\\ & \rho_{\text{yd}2}=0.4661508,\;\rho_{d1d2}=0.9153857,\;C_{d1}=1.174456,\;C_{d2}=1.283018,\;C_{y}=2.341245 \end{array}$$

**Population 4.** (Source from agricultural sector)

Let y represent the amount of cotton produced in hectares in 2009, $d_{1}$ represent the percentage of farms with cotton cultivation areas greater than 37 ha in 2007, and $d_{2}$ represent the percentage of farms with cotton cultivation areas greater than 35 ha in 2008 for 52 districts in Pakistan. The population information about data set is given as:

$$\begin{array}{cc} & N=52,\;n=11,\;\bar{Y}=50.03846,\;D_{1}=0.3846154,\;D_{2}=0.4423077,\;\rho_{\text{yd}1}=0.7369579,\\ & \;\rho_{\text{yd}2}=0.6935718,\;\rho_{d1d2}=0.8877181,\;C_{d1}=1.277252,\;C_{d2}=1.13384,\\ & \;C_{y}=1.421524 \end{array}$$

We compute the MSE values of existing estimates and proposed estimator using Eqs. (2), (4), (6), (8), (10), (12), (14), (16), (18) and (24) and these values are shown in Table 1.

**Table 1:** MSE values estimators.

Population				
Estimator	$P_{1}$	$P_{2}$	$P_{3}$	$P_{4}$
${\hat{\mu }}_{0}$	2515.170	2,115,180.565	435,363.127	362.664
${\hat{\mu }}_{1}$	1665.169	1,787,076.238	352,906.039	175.164
${\hat{\mu }}_{2}$	9050.198	3,274,574.672	736,929.821	1135.734
${\hat{\mu }}_{3}$	1379.541	1,847,217.179	366,745.882	195.717
${\hat{\mu }}_{4}$	2536.028	1,295,483.198	279,378.887	338.001
${\hat{\mu }}_{5}$	1315.997	1,782,466.925	351,230.425	165.699
${\hat{\mu }}_{6}$	5151.870	2,045,097.266	480,329.895	466.088
${\hat{\mu }}_{7}$	10,154.721	4,937,999.787	870,994.957	1306.264
${\hat{\mu }}_{8}$	1275.439	1,496,047.750	340,313.804	163.048
${\hat{\mu }}_{prop}$	**1200.370**	**1,290,907.767**	**206,924.055**	**106.485**

Note: Bold number indicate the lowest MSE.

To measure the Percentage Relative Efficiency (PRE), we apply the following formula:

$$PRE=\frac{MSE\left({\hat{\mu }}_{0}\right)}{MSE\left(t\right)\;}\;X\;100$$

where *t=*${\hat{\mu }}_{0},{\hat{\mu }}_{1},{\hat{\mu }}_{2},{\hat{\mu }}_{3},{\hat{\mu }}_{4},{\hat{\mu }}_{5},{\hat{\mu }}_{6},{\hat{\mu }}_{7},{\hat{\mu }}_{8},\;$and ${\hat{\mu }}_{prop}$

The numerical comparison of PRE for existing and proposed estimator is shown in Table 2.

**Table 2:** Percentage Relative Efficiency (PRE) of estimators.

Population				
Estimator	$P_{1}$	$P_{2}$	$P_{3}$	$P_{4}$
${\hat{\mu }}_{0}$	100	100	100	100
${\hat{\mu }}_{1}$	151.046	118.360	123.365	207.043
${\hat{\mu }}_{2}$	27.791	64.594	59.078	31.932
${\hat{\mu }}_{3}$	182.319	114.506	118.710	185.300
${\hat{\mu }}_{4}$	99.178	163.273	155.833	107.297
${\hat{\mu }}_{5}$	191.123	118.666	123.954	218.870
${\hat{\mu }}_{6}$	48.821	103.427	90.638	77.810
${\hat{\mu }}_{7}$	24.768	42.835	49.985	27.763
${\hat{\mu }}_{8}$	163.048	141.384	127.929	222.428
${\hat{\mu }}_{prop}$	**209.533**	**163.852**	**210.398**	**340.578**

Note: Bold number indicate the higest PRE.

**Fig. 2:**
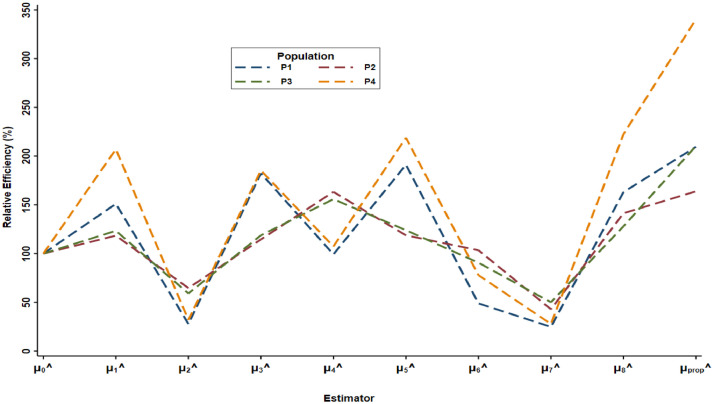
Graphical representation of PRE of estimatotrs w.r.t to ${\hat{\mu }}_{0}$.

The proposed estimator is compared with the eight estimators as shown in Section 2 and results shown numerically and graphically. It is observed from Table 1, that proposed estimator attains the minimum mean square error 1200.370, 1,290,907.767, 206,924.055 and 106.485 for populations $\left(P_{1},\ldots ,P_{4}\right)$, respectively. While from Table 2, it is revealed that PRE value of the proposed estimator for all four populations ($P_{1},..,P_{4}$) is 209.533, 163.852, 210.398 and 340.578, respectively. The PRE of proposed estimator is highest in comparison to the existing estimators as well as all the estimators given in Ahmed et al. [8]. It is observed from Figs. 1 and 2 that proposed estimator is more efficient for population mean estimation in comparison to the existing estimators. In theory of sample surveys, the major objective of any statistician or investigator is to minimize the MSE and maximize the PRE in the estimation to ideally draw an inference about the study population. The proposed estimator is validated with the help of minimum MSE and maximum PRE. It attains the minimum MSE and maximum PRE in comparison of the other existing estimators as shown graphically for all four populations.

## Conclusion

In this article, we proposed an optimum estimator for estimation of population mean in simple random sampling by utilizing the information of the two auxiliary attribute. Mathematical expressions of the proposed estimator i.e. bias, mean squared error (MSE) and minimum mean squared error are derived. Mean square error of proposed estimator is shown in Section 3. The proposed estimator is efficient under the derived conditions discussed in Section 4. The implementation of proposed estimator helps in estimation of précised value of the population mean on the basis of which effective decisions can be made. It is recommended that the proposed estimator may be used in sample surveys in the areas of education, agriculture fisheries and health sciences, etc. Further, the proposed estimator can be extended to utilization of multi auxiliary information under various sampling designs.

## CRediT authorship contribution statement

**Monika Saini:** Conceptualization, Methodology, Validation, Writing – review & editing. **Bhatt Ravi Jitendrakumar:** Investigation, Resources, Writing – original draft. **Ashish Kumar:** Formal analysis, Methodology, Writing – review & editing.

## Declaration of Competing Interest

There is no conflict of interest among the authors.

## Figures and Tables

**Table:** 

Hypothetical Population: (Source: Singh and Choudhary [19], pp. 177)			
S.N.	Area under wheat (in acres)		
	(1971)	(1973)	(1974)
	(x_1_)	(x_2_)	(y)
1	401	70	50
2	630	163	149
3	1194	320	284
4	1170	440	381
5	1065	250	278
6	827	125	111
7	1737	558	634
8	1060	254	278
9	360	101	112
10	946	359	355
11	4170	109	99
12	1625	481	498
13	827	125	111
14	96	5	6
15	1304	427	339
16	377	78	80
17	259	75	105
18	186	45	27
19	1767	564	515
20	604	238	249
21	700	92	85
22	524	247	221
23	571	134	133
24	962	131	144
25	407	129	103
26	715	190	175
27	845	363	335
28	1016	235	219
29	184	73	62
30	282	62	79
31	194	71	60
32	439	137	100
33	854	196	141
34	820	255	263

## Data Availability

Data is available in public domain.

## Appendix A

Hypothetical Population: (Source: Singh and Choudhary (20), pp. 177)

Let y be the area under wheat crop (in acres) during the year 1974, x_1_ be the area under wheat (in acres) 1971 and x_2_ be the area under wheat (in acres) in 1973.

The population information about data set is given as:

ẟ_1_ = 1 if farms under the wheat crop which have more than 500 acres land during the year 1971, otherwise ẟ_1_ = 0.
ẟ_2_ = 1 if farms under wheat crop which have more than 100 acres land during the year 1973, otherwise ẟ_2_ = 0.
$D_{1}$ be the proportion of farms under the wheat crop which have more than 500 acres land during the year 1971 and $D_{2}$ be the proportion of farms under wheat crop which have more than 100 acres land during the year 1973.

Descriptive Statistics:

$$\begin{array}{cc} N=34, & \;\bar{\;Y}=199.4,\;D_{1}=0.6765,\;D_{2}=0.7353,\;\rho_{\text{yd}1}=0.599,\;\rho_{\text{yd}2}=0.599,\\ & \;\rho_{d1d2}=0.725,\;\;S_{d1}^{2}=0.225490,\;\;S_{d2}^{2}=0.200535,\;S_{y}^{2}=22,564.6 \end{array}$$
